# Enterovirus Replication and Dissemination Are Differentially Controlled by Type I and III Interferons in the Gastrointestinal Tract

**DOI:** 10.1128/mbio.00443-22

**Published:** 2022-05-23

**Authors:** Alexandra I. Wells, Kalena A. Grimes, Carolyn B. Coyne

**Affiliations:** a Department of Molecular Genetics and Microbiology, Duke University School of Medicine, Durham, North Carolina, USA; b Duke Human Vaccine Institute, Duke University School of Medicine, Durham, North Carolina, USA; St. Jude Children’s Research Hospital

**Keywords:** GI tract, enterovirus, interferons

## Abstract

Enteroviruses are among the most common viral infectious agents of humans and cause a broad spectrum of mild-to-severe illness. Enteroviruses are transmitted primarily by the fecal-oral route, but the events associated with their intestinal replication *in vivo* are poorly defined. Here, we developed a neonatal mouse model of enterovirus infection by the enteral route using echovirus 5 and used this model to define the differential roles of type I and III interferons (IFNs) in enterovirus replication in the intestinal epithelium and subsequent dissemination to secondary tissues. We show that human neonatal Fc receptor (FcRn), the primary receptor for echoviruses, is essential for intestinal infection by the enteral route and that type I IFNs control dissemination to secondary sites, including the liver. In contrast, type III IFNs limit echovirus infection in the intestinal epithelium, and mice lacking this pathway exhibit extended epithelial replication. Finally, we show that echovirus infection in the small intestine is cell type specific and occurs exclusively in enterocytes. These studies define the type-specific roles of IFNs in enterovirus infection of the gastrointestinal (GI) tract and the cellular tropism of echovirus replication in the intestinal epithelium.

## INTRODUCTION

Enteroviruses are small (~30-nm) single-stranded RNA viruses that are comprised of coxsackieviruses (coxsackievirus A [CVA] and CVB), rhinoviruses, poliovirus (PV), enteroviruses 71 and D68 (EV71 and EVD68, respectively), and echoviruses (which include ~30 serotypes). Echoviruses cause 15 to 30% of nosocomial infections in neonatal intensive care units (NICUs) and often result in aseptic meningitis and liver failure, which can be fatal (1–4). The National Enterovirus Surveillance System (NESS) indicates that between 2014 and 2016, echoviruses were among the most commonly circulating enteroviruses in the United States (5). Globally, outbreaks of echoviruses, including echovirus 5 (E5), have been associated with a range of clinical outcomes, with the most severe disease occurring in infants and children (6–8).

Enteroviruses are transmitted primarily through the fecal-oral route and initiate host entry through the epithelial lining of the gastrointestinal (GI) tract. We have previously shown that echoviruses robustly infect human stem cell-derived intestinal enteroids and exhibit cell type specificity of infection, with preferential infection in enterocytes and enteroendocrine cells (9, 10). Additionally, echovirus infections cause damage to barrier function in enteroid-derived intestinal epithelial monolayer cultures (10), suggesting that virus-mediated epithelial damage could contribute to dissemination from the intestine. The impact of host innate immune signaling on enteroviral infections in the intestinal epithelium is largely unknown. Previous studies in mouse models using PV, CVB, and EV71 have shown that the ablation of type I interferon (IFN) signaling by the deletion of the IFN-α/β receptor (IFNAR) is required for infection by the oral route (11–16), suggesting that these IFNs play a central role in the protection of the GI tract from enterovirus infection. Whether this enhancement was the result of increased infection in the intestinal epithelium directly and/or resulted from alterations in infection of nonepithelial cell types remains unclear. Type III IFNs, which are comprised of IFN-λs 1 to 3 in humans, are preferentially induced in enterovirus-infected human enteroids (9, 10). For other enteric viruses such as reoviruses, rotaviruses, and noroviruses, type III IFNs specifically control viral replication in the intestinal epithelium *in vivo*, with type I IFNs impacting the lamina propria (11, 17–20). Thus, the roles of type I and III IFNs in the control of enteroviral infections *in vivo* remain unclear, as does whether these IFNs function in a type-specific manner to control enterovirus infections in the GI tract.

We and others previously identified the human neonatal Fc receptor (hFcRn) as a primary receptor for echoviruses (21, 22). In contrast, mouse FcRn does not function as an echovirus receptor and does not support replication *in vivo* (21, 23). FcRn is expressed at the apical membrane of enterocytes, where it binds to IgG, is internalized by endocytosis, and delivers its cargo to early and late endosomes, with the eventual release of IgG into the interstitium (24). However, the expression of hFcRn alone is not sufficient for echovirus 11 (E11) infection in adult or neonatal mice, and ablation of IFNAR in hFcRn-expressing mice is required for infection following intraperitoneal (i.p.) inoculation (23). However, the roles of hFcRn and IFN signaling following inoculation via the enteral route were not explored.

Here, we established *in vitro* and *in vivo* models to define the impact of hFcRn expression and type I and III IFN signaling on echovirus infections of the GI epithelium. To do this, we generated mice expressing hFcRn that are deficient in the type III IFN receptor (IFNLR) and compared their susceptibility to oral echovirus infection to that of hFcRn-expressing mice lacking IFNAR expression or immunocompetent animals expressing hFcRn alone. Given that some echoviruses, including E11, utilize decay-accelerating factor (DAF/CD55) as an attachment factor and that the impact of these receptors on pathogenesis is unknown, we performed these studies using E5, which does not bind DAF (21, 25). Whereas the expression of hFcRn was necessary and sufficient to support echovirus replication in primary murine stem cell-derived enteroids, it was not sufficient for infection of immunocompetent mice following oral gavage. We show that hFcRn-expressing mice deficient in IFNLR expression poorly control echovirus infection in the GI tract and exhibit sustained replication in the intestinal epithelium, which occurred exclusively in enterocytes. However, these animals did not exhibit any morbidity or mortality, and there was no dissemination to secondary tissues such as the liver or pancreas. In contrast, there was robust dissemination of E5 following oral gavage of hFcRn-expressing mice deficient in IFNAR expression, which resulted in significant morbidity and mortality. However, we did not observe active replication in the intestinal epithelium of these animals. These findings define the differential roles of type I and III IFNs in the control of echovirus replication in the GI tract and subsequent dissemination to secondary sites of infection.

## RESULTS

### Human FcRn is required for echovirus infection of murine-derived enteroids.

**FIG 1:**
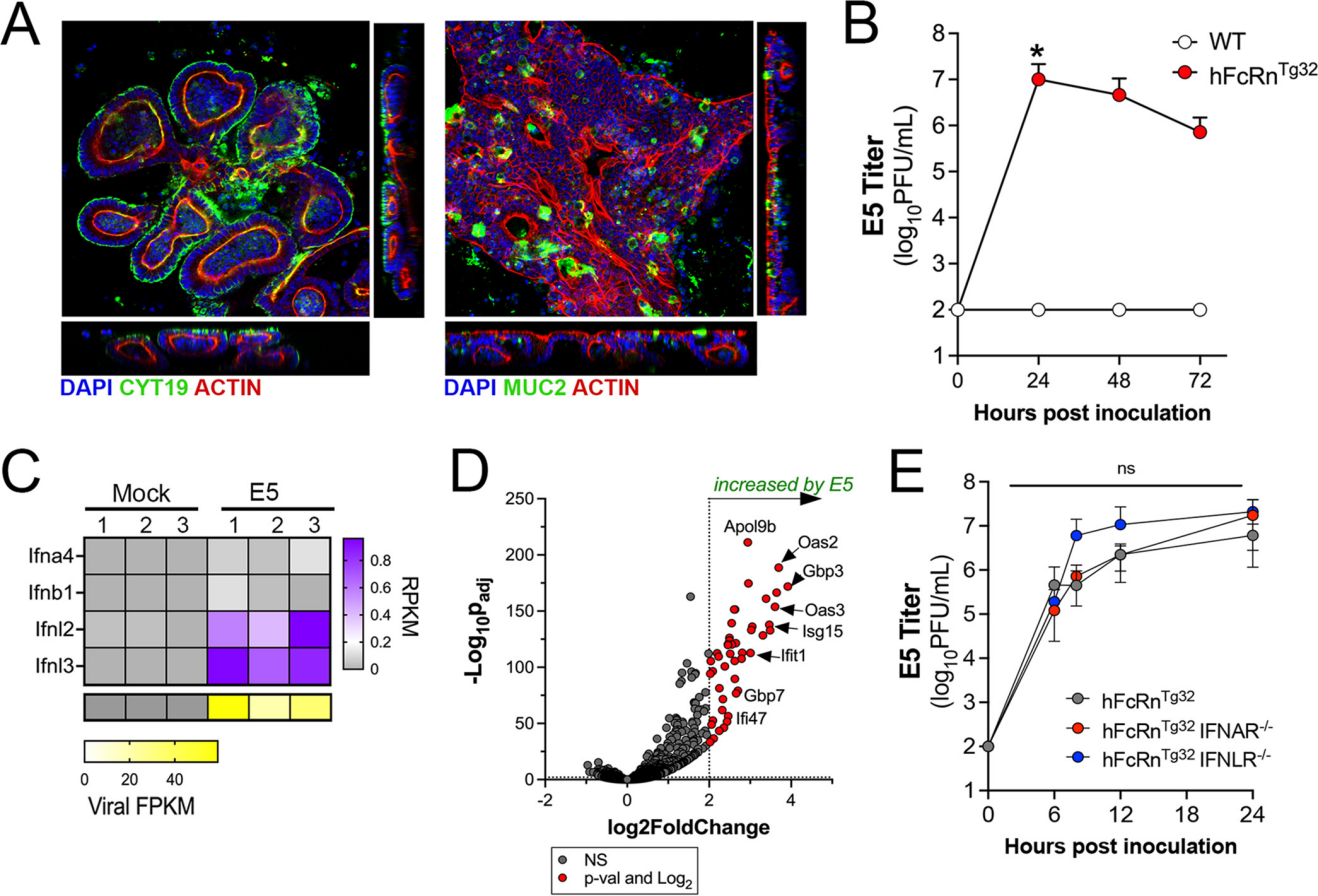
Human FcRn is necessary and sufficient for echovirus infection of murine-derived primary enteroids. (A) Murine enteroids were generated from Lgr5^+^ crypts isolated from the small intestines of five 10-day-old neonatal C57BL/6J (WT) mice. Confocal microscopy images of enteroids immunostained with cytokeratin-19 in green and actin in red (left) or mucin-2 in green and actin in red (right) ~10 days after culturing are shown. (B) WT (white) or hFcRn^Tg32^ (red) enteroids were generated from small intestine tissue from 10-day-old neonatal mice and infected with 10^6^ PFU of neutral red-incorporated E5. Viral titers (log_10_ PFU per milliliter) were assessed in the cell culture supernatants at the indicated time points. (C) Heatmap of reads per kilobase per million (RPKM) values of the type I IFNs Ifna4 and Ifnb1 and the type III IFNs Ifnl2 and Ifnl3 from bulk RNASeq of uninfected (mock) or E5-infected hFcRn^Tg32^ enteroids at 24 h postinfection. In the key at the right, purple indicates higher reads, and gray denotes no reads detected. At the bottom are viral fragments per kilobase per million (FPKM) values from the samples shown at the top. Yellow indicates high viral RNA reads, and gray denotes no reads detected. (D) Volcano plot comparing differentially expressed transcripts in E5-infected hFcRn^Tg32^ enteroids compared to mock controls as determined by DESeq2 analysis. Gray circles represent genes whose expression was not significantly changed (NS). Red circles represent genes that were significantly changed by E5 infection. Significance was set at a *P* value of <0.01 and a log_2_ fold change of ±2. (E) Enteroids generated from the small intestine of hFcRn^Tg32^ (gray), hFcRn^Tg32^-IFNAR^−/−^ (red), or hFcRn^Tg32^-IFNLR^−/−^ (blue) mice were infected with 10^6^ PFU of neutral red-incorporated E5. Viral titers (log_10_ PFU per milliliter) are shown at the indicated time points. In panels B and E, data are shown as means ± standard deviations from three independent replicates. Enteroids were isolated from at least five 10-day-old neonatal mice and pooled during Lgr5^+^ crypt isolation. Significance in panel B was determined by a Kruskal-Wallis test with Dunn’s test for multiple comparisons. Significance in panel D was determined using two-way ANOVA with a Geisser-Greenhouse correction and Tukey’s multiple-comparison test (*, *P* < 0.05; ns, not significant).

To define the role of hFcRn in infections of the murine intestine, we generated neonatal enteroids from C57BL/6 (wild-type [WT]) mice and mice expressing hFcRn (hFcRn^Tg32^). hFcRn^Tg32^ mice are deficient in the expression of mouse FcRn and express human FcRn under the control of the native human promoter (26). Stem cell-derived enteroids differentiated to form three-dimensional structures containing cells present in the epithelium *in vivo*, including enterocytes and mucin-secreting goblet cells (Fig. 1A). Consistent with what has been described previously for murine fibroblasts derived from WT mice (21), enteroids derived from WT mice were resistant to E5 infection (Fig. 1B). In contrast, enteroids derived from hFcRn^Tg32^ mice were highly permissive to E5 infection, which peaked at ~24 h postinoculation (hpi) (Fig. 1B). To define the host response of enteroids to E5 infection, we performed bulk RNA sequencing (RNASeq) followed by differential expression analysis. Similar to previously reported results in human enteroids (9), murine-derived enteroids induced the selective expression of transcripts associated with the type III IFNs IFN-λ-2 and IFN-λ-3 (mice do not express IFN-λ-1) (Fig. 1C). Differential expression analysis revealed the induction of 48 transcripts in E5-infected hFcRn^Tg32^ enteroids, 42 of which are classified as interferon-stimulated genes (ISGs) (Fig. 1D), supporting a prominent role of IFN signaling in the intestinal innate immune response to echovirus infections.

The selective induction of type III IFNs in murine-derived enteroids suggests that these IFNs are key mediators in the control of echovirus infections in the intestinal epithelium. To test this, we derived enteroids from small intestine tissue of mice expressing hFcRn that are deficient in IFNAR expression (hFcRn^Tg32^-IFNAR^−/−^) (23). To perform parallel studies in enteroids deficient in type III IFN signaling, we crossed hFcRn^Tg32^ mice to mice deficient in IFNLR expression (hFcRn^Tg32^-IFNLR^−/−^). Enteroids were generated from the small intestines of immunocompetent hFcRn^Tg32^, hFcRn^Tg32^-IFNAR^−/−^, and hFcRn^Tg32^-IFNLR^−/−^ mice, and the levels of E5 replication were compared among these genotypes. We did not detect any significant differences in E5 replication between hFcRn-expressing immunocompetent enteroids and those deficient in either IFNAR or IFNLR expression (Fig. 1E). These data show that in *ex vivo* murine-derived enteroid models, hFcRn expression is necessary and sufficient for echovirus infection of the intestinal epithelium.

### Human FcRn is necessary but not sufficient for echovirus infection of the intestine *in vivo*.

**FIG 2:**
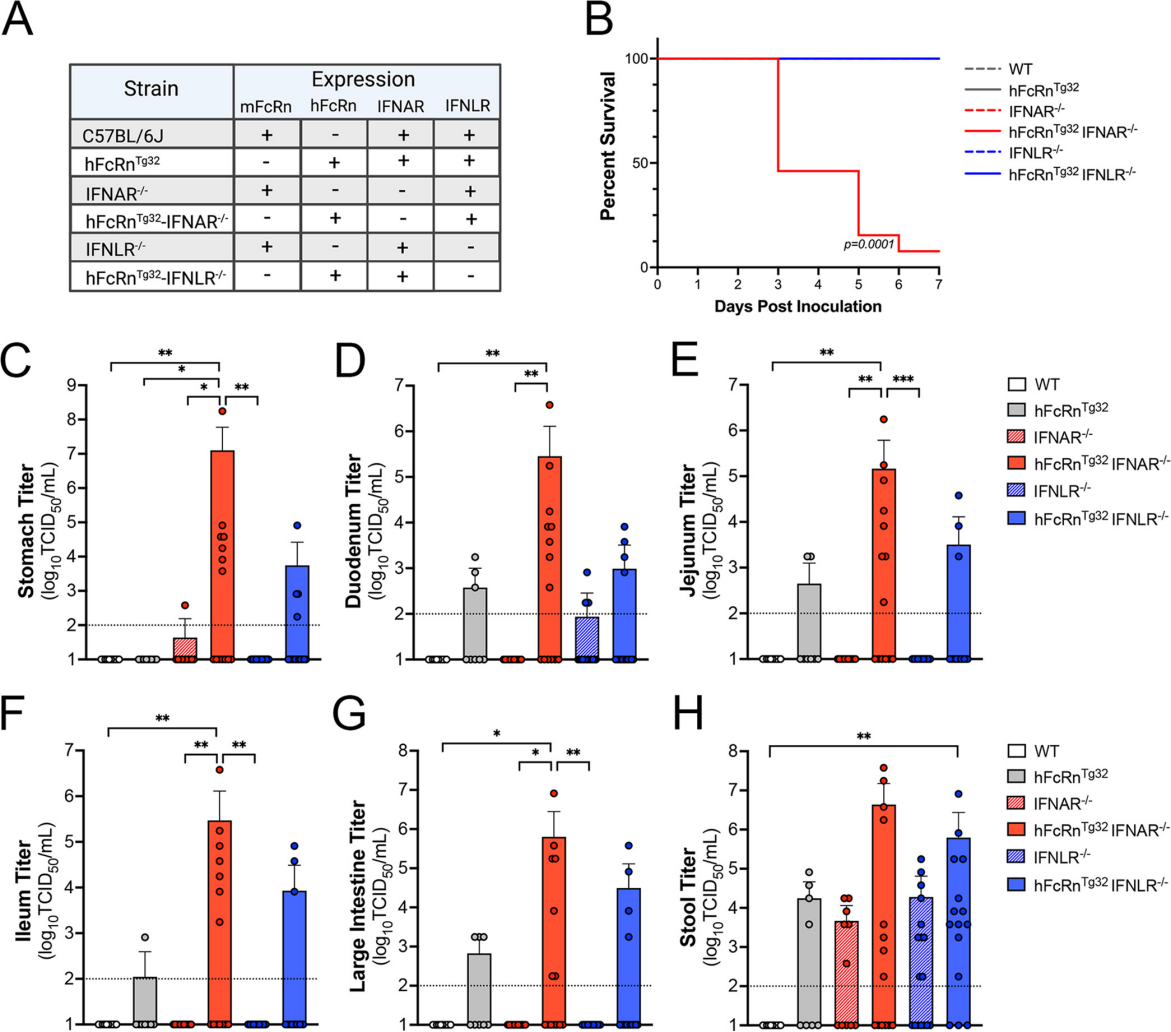
Expression of human FcRn is not sufficient for echovirus infection by the enteral route *in vivo*. (A) Table of the six genotypes used in this study. Shown is the expression of mouse or human FcRn, IFNAR, and IFNLR among these genotypes. (B) Survival of the indicated genotype of mice inoculated with 10^6^ PFU of E5 by oral gavage for 7 days postinoculation. The log rank test was used to analyze the statistical differences of the survival rates. (C to H) At 3 dpi, animals were sacrificed, and viral titers in the stomach (C), duodenum (D), jejunum (E), ileum (F), large intestine (G), and stool (H) were determined by TCID_50_ assays. In all panels, titers are shown as log_10_ TCID_50_ per milliliter, with the limit of detection indicated by a dotted line. Data are shown as means ± standard deviations, with individual animals shown as each data point. Data are shown with significance determined by a Kruskal-Wallis test with Dunn’s test for multiple comparisons (*, *P* < 0.05; **, *P* < 0.005; ***, *P* < 0.0005).

As described above, *ex vivo* enteroid models suggested that echovirus infection of murine-derived intestinal cells depended on the expression of hFcRn and was not controlled by either type I or III IFNs. However, enteroids may not fully recapitulate the events associated with infection *in vivo*. To address this, we used six genotypes of mice, including the humanized FcRn models described above (hFcRn^Tg32^, hFcRn^Tg32^-IFNAR^−/−^, and hFcRn^Tg32^-IFNLR^−/−^) and animals expressing murine FcRn that were immunocompetent (C57BL/6 [WT]) or deficient in type I or III IFN signaling (IFNAR^−/−^ or IFNLR^−/−^, respectively) (Fig. 2A). Neonatal (7-day-old) mice were orally inoculated with 10^6^ PFU of E5 and monitored daily for 7 days for signs of illness (e.g., inactivity, discoloration, lack of nursing, lack of parental care, and death). We observed death in approximately 50% of hFcRn^Tg32^-IFNAR^−/−^ animals by 3 days postinoculation (dpi) and almost 100% lethality by 7 dpi (Fig. 2B). In contrast, there were no clinical symptoms of illness in any other genotype, and all animals survived until 7 dpi (Fig. 2B). There were no significant differences in mortality between male and female hFcRn^Tg32^-IFNAR^−/−^ mice (see Fig. S1A in the supplemental material).

10.1128/mbio.00443-22.1 FIG S1 Seven-day-old pups were orally inoculated with 10^6^ PFU of E5. (A) Survival curve of hFcRn^Tg32^-IFNAR^−/−^ animals broken down by sex. A log rank test was used to analyze the statistical differences of the survival rates. (B) Viral titers of hFcRn^Tg32^-IFNAR^−/−^ animals at 3 dpi broken down by sex. (C) Viral titers of hFcRn^Tg32^-IFNLR^−/−^ animals at 7 dpi broken down by sex. Significance was determined by a Mann-Whitney U test (*P* values shown). Each symbol represents an individual animal. Download FIG S1, TIF file, 0.7 MB. Copyright © 2022 Wells et al. 2022 Wells et al. https://creativecommons.org/licenses/by/4.0/ This content is distributed under the terms of the Creative Commons Attribution 4.0 International license.

We next determined the extent of viral replication in the GI tract of infected animals at 3 dpi by measuring viral titers in the stomach, small intestine (duodenum, jejunum, and ileum), large intestine, and stool. Infected hFcRn^Tg32^-IFNAR^−/−^ animals contained high levels of virus in all tissues collected, with half or more of the animals having high viral loads in the stomach (7 of 14 mice), duodenum (8 of 14 mice), jejunum (8 of 14 mice), ileum (7 of 14 mice), and large intestine (7 of 14 mice) (Fig. 2C to H). In contrast, there was no detectable virus in most tissues isolated from mice not expressing hFcRn, including WT (0 of 10 mice), IFNAR^−/−^ (0 of 11 mice), and IFNLR^−/−^ (0 of 17 mice) (Fig. 2C to H). Three IFNLR^−/−^ mice had detectable virus in the duodenum; however, this was not statistically significant (Fig. 2D). There were low levels of virus detected in select tissues from immunocompetent hFcRn^Tg32^ animals, which included the duodenum (3 of 8 mice), jejunum (2 of 8 mice), ileum (1 of 8 mice), and large intestine (3 of 8 mice). No virus was recovered from the stomachs of hFcRn^Tg32^ mice (0 of 8 mice). Similarly, low- to mid-level titers were observed in hFcRn^Tg32^-IFNLR^−/−^ mice, with virus being recovered from the stomach (3 of 15 mice), duodenum (4 of 15 mice), jejunum (3 of 15 mice), ileum (3 of 15 mice), and large intestine (4 of 15 mice). Stool samples collected from all genotypes except the WT contained high levels of virus in the stool, which may reflect the remaining inoculum. There were no significant differences in titers between male and female hFcRn^Tg32^-IFNAR^−/−^ mice, although male mice did have overall higher titers in various regions of the small intestine (Fig. S1B). These data show that hFcRn is necessary, but not sufficient, for echovirus infection of the intestine *in vivo* and that type I and III IFNs differentially control replication and pathogenesis.

### Type I IFNs are the primary drivers of dissemination outside the GI tract.

**FIG 3:**
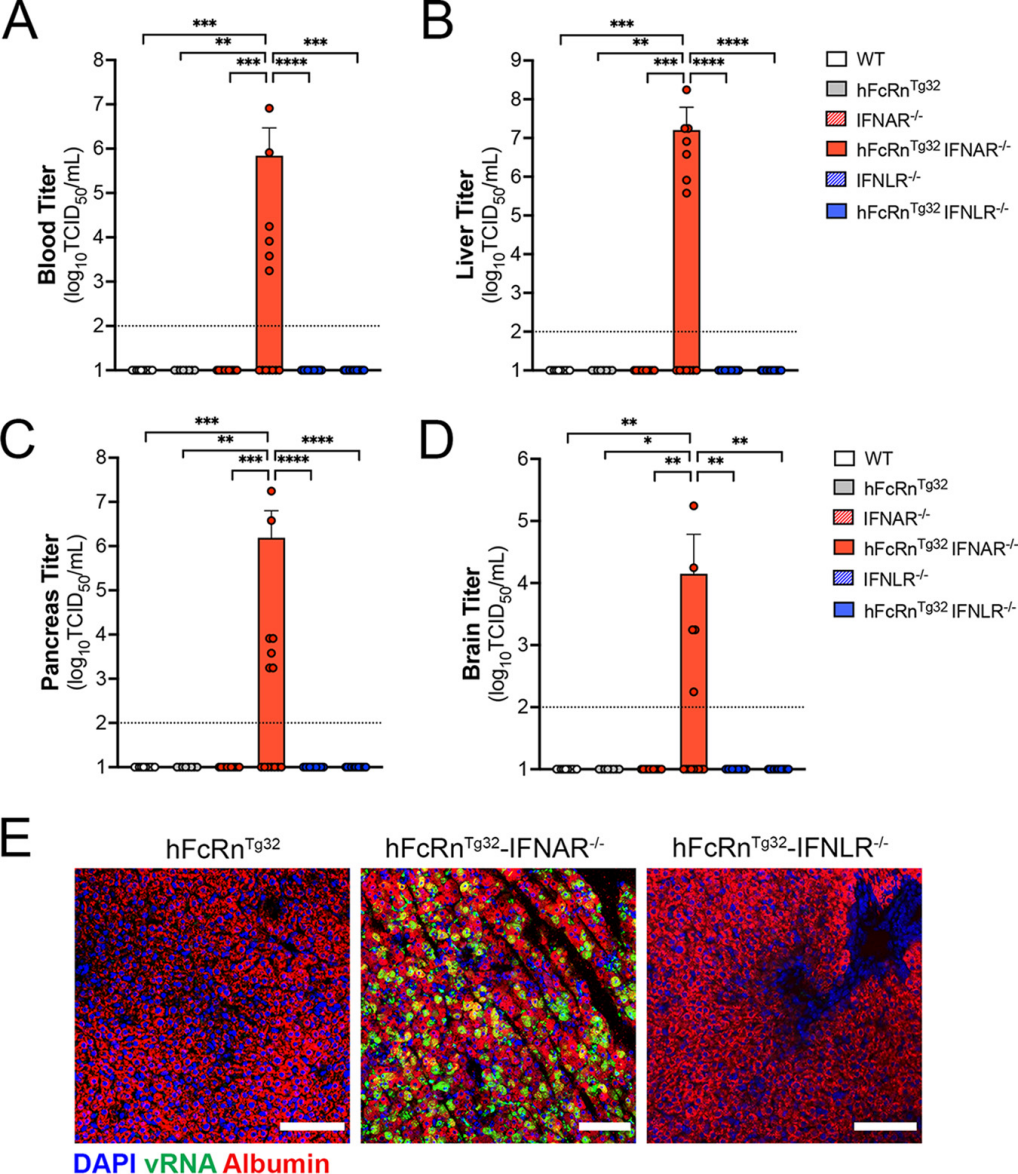
Type I IFNs control echovirus dissemination from the GI tract. Seven-day-old pups were orally inoculated with 10^6^ PFU of E5, and at 3 dpi, animals were sacrificed for viral titration and histology. (A to D) Viral titers in the blood (A), liver (B), pancreas (C), and brain (D). In all panels, titers are shown as log_10_ TCID_50_ per milliliter, with the limit of detection indicated by a dotted line. Data are shown as means ± standard deviations, with individual animals shown as each data point. (E) Hybridization chain reaction (HCR) RNA fluorescence *in situ* hybridization (RNA-FISH) from liver sections of hFcRn^Tg32^, hFcRn^Tg32^-IFNAR^−/−^, or hFcRn^Tg32^-IFNLR^−/−^ neonatal mice at 3 dpi using probes against the E5 genome (green) and albumin (red). DAPI-stained nuclei are shown in blue. Bars, 100 μm. In panels A to D, data are shown with significance determined by a Kruskal-Wallis test with Dunn’s test for multiple comparisons (*, *P* < 0.05; **, *P* < 0.005; ***, *P* < 0.0005; ****, *P* < 0.0001).

Given the high degree of mortality in orally inoculated hFcRn^Tg32^-IFNAR^−/−^ mice, we next assessed the levels of infection at key secondary sites of infection at 3 dpi, including the liver, pancreas, and brain, which are all targeted by echoviruses in humans. hFcRn^Tg32^-IFNAR^−/−^ mice had higher levels of circulating virus (6 of 13 mice), which was not detected in any other genotype (Fig. 3A). Consistent with this, we did not detect any virus in the livers, pancreases, or brains of WT, hFcRn^Tg32^, IFNAR^−/−^, IFNLR^−/−^, or hFcRn^Tg32^-IFNLR^−/−^ animals (Fig. 3B to D). In contrast, hFcRn^Tg32^-IFNAR^−/−^ mice contained very high titers in the liver (7 of 14 mice) and pancreas (7 of 14 mice) and lower titers in the brain (5 of 14 mice) (Fig. 3B to D). There were no significant differences in titers between male and female mice, although male mice did have overall higher titers in the liver (Fig. S1B). These data are consistent with our previously reported data showing that hFcRn^Tg32^-IFNAR^−/−^ pups or adult mice inoculated by the i.p. route have high levels of echovirus infection in the liver and pancreas (23).

Next, we performed Luminex multiplex assays to determine the levels of 25 circulating cytokines in the blood of E5-infected animals. Consistent with their high levels of dissemination, we found that hFcRn^Tg32^-IFNAR^−/−^ mice induced pronounced antiviral and proinflammatory signaling in response to E5 infection, which included high levels of circulating type I IFNs (IFN-α and IFN-β), granulocyte colony-stimulating factor (G-CSF), and interleukin-6 (IL-6) (Fig. S2A to D). No other genotypes contained any significant increases in circulating cytokines (Fig. S2A to D). These data are similar to those of our previous work where i.p. inoculated hFcRn^Tg32^-IFNAR^−/−^ animals had high levels of circulating type I IFNs (23).

10.1128/mbio.00443-22.2 FIG S2 Neonatal mice were inoculated by the oral route with 10^6^ PFU of E5 and sacrificed at 3 dpi. Luminex-based multianalyte profiling of 26 cytokines was then performed from whole blood. (A) Heatmap demonstrating the induction (shown as fold changes from the uninfected control) in E5-infected mice of the indicated genotype. Blue denotes significantly increased cytokines in comparison to the untreated controls. Gray or white denotes little to no change (scale at the top right). (B to D) IFNs are shown to the right as picograms per milliliter of IFN-α (B), IFN-β (C), and IFN-λ-2/3 (D). Data are shown as means ± standard deviations and individual animals (points). Data are shown with significance determined by a Kruskal-Wallis test with Dunn’s test for multiple comparisons (*, *P* < 0.05; **, *P* < 0.005; ns, not significant). Each symbol represents an individual animal. Download FIG S2, TIF file, 1.0 MB. Copyright © 2022 Wells et al. 2022 Wells et al. https://creativecommons.org/licenses/by/4.0/ This content is distributed under the terms of the Creative Commons Attribution 4.0 International license.

Because we observed significant dissemination of E5 to the livers of orally inoculated hFcRn^Tg32^-IFNAR^−/−^ mice, we next determined if the cellular tropism of echoviruses is the same between the i.p. and oral routes of inoculation. To do this, we performed a hybridization chain reaction (HCR), which allows multiplexed fluorescent quantitative RNA detection with enhanced sensitivity over conventional hybridization approaches (27, 28). Our previous work using this method showed that echoviruses exclusively target hepatocytes following i.p. inoculation (23). We designed probes specific for the E5 genome and used probes to the hepatocyte marker albumin, and we performed HCR on liver sections from hFcRn^Tg32^, hFcRn^Tg32^-IFNAR^−/−^, and hFcRn^Tg32^-IFNLR^−/−^ mice orally inoculated with E5 at 3 dpi. E5 viral RNA (vRNA)-positive cells colocalized exclusively with albumin, identifying hepatocytes as the main cellular target of infection in the liver following dissemination from the GI tract (Fig. 3E). Collectively, these data show that type I IFNs are the primary drivers of echovirus dissemination from the GI tract to secondary sites, including the liver and pancreas.

### Type III IFNs limit persistent echovirus infection in the GI epithelium.

**FIG 4:**
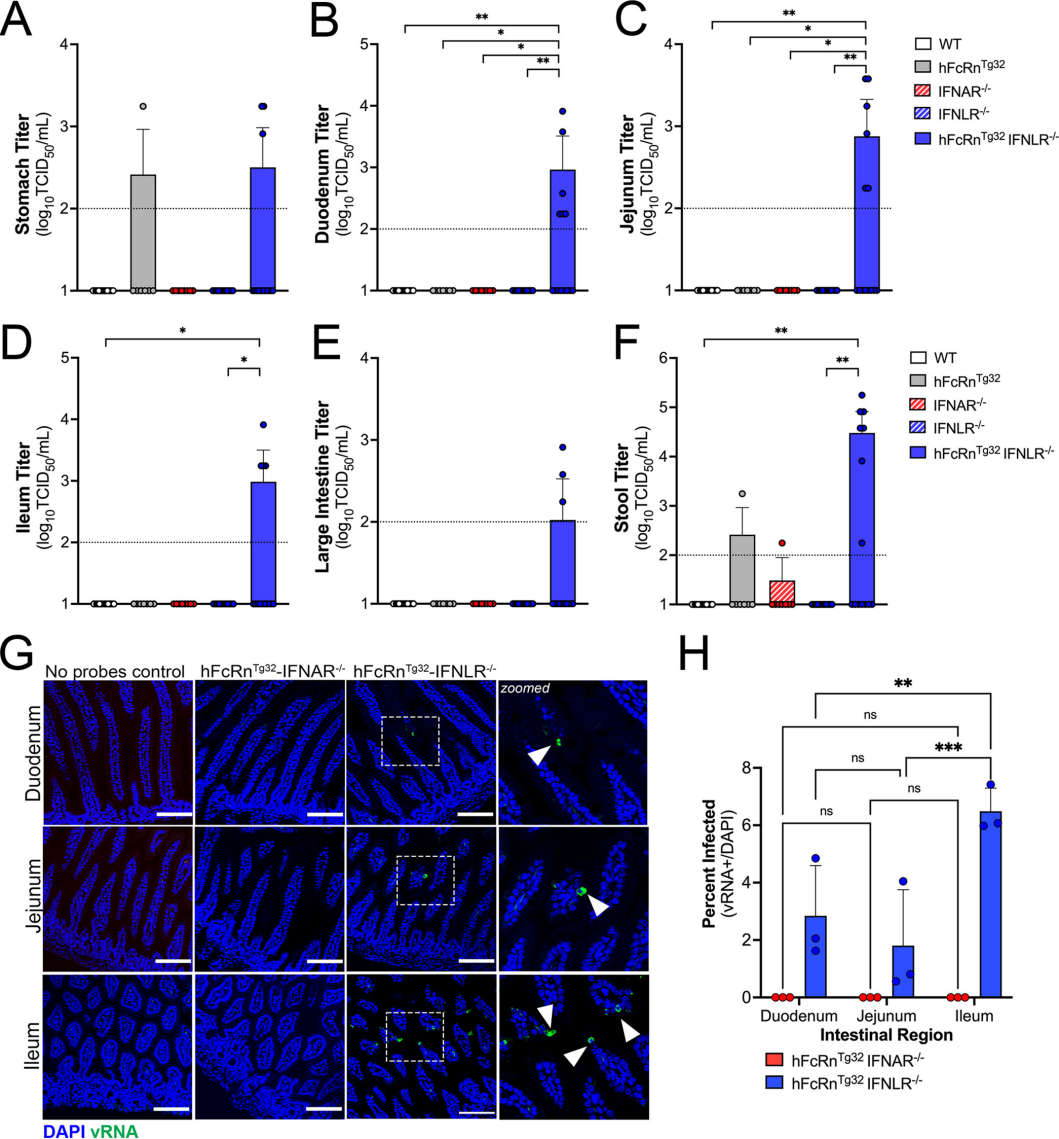
Type III IFNs restrict persistent echovirus infection in the GI epithelium. Seven-day-old neonatal mice were orally inoculated with 10^6^ PFU of E5, and at 7 dpi, animals were sacrificed for viral titration and tissue collection. (A to F), Viral titers in the stomach (A), duodenum (B), jejunum (C), ileum (D), large intestine (E), and stool (F). In all panels, titers are shown as log_10_ TCID_50_ per milliliter, with the limit of detection indicated by a dotted line. Data are shown as means ± standard deviations, with individual animals shown as each data point. Significance was determined using a Kruskal-Wallis test with Dunn’s test for multiple comparisons (*, *P* < 0.05; **, *P* < 0.005). (G) At 3 dpi, animals were sacrificed, and the entire GI tract was removed and Swiss rolled, followed by histological sectioning. HCR of hFcRn^Tg32^-IFNAR^−/−^ or hFcRn^Tg32^-IFNLR^−/−^ pups at 3 dpi was performed using probes against the E5 genome (green) and DAPI (blue). Bars, 100 μm. Zoomed-in views of specific regions of the hFcRn^Tg32^-IFNLR^−/−^ images are shown on the right. (H) Quantification of three independent tile scans using confocal microscopy of each region of the small intestine based on the number of villi that were positive for vRNA using the cell count function in FIJI. Data are shown as a percentage of vRNA-positive villi over total villi per tile scan. Three independent tile scans were quantified (for an average of 144 villi in the duodenum, 224 villi in the jejunum, and 164 villi in the ileum). Significance was determined by two-way ANOVA with Šídák’s multiple-comparison tests (*, *P* < 0.05; **, *P* < 0.005; ***, *P* < 0.0005; ****, *P* < 0.0001).

Because we observed low levels of E5 replication in GI-derived tissues at 3 dpi, we next compared viral titers from tissues isolated at 7 dpi to determine if there were differences in persistence compared to hFcRn^Tg32^-IFNAR^−/−^ mice. As hFcRn^Tg32^-IFNAR^−/−^ mice died from disease before 7 dpi, they were excluded from these studies. At 7 dpi, hFcRn^Tg32^-IFNLR^−/−^ mice were the only genotype with consistently detectable virus in tissues associated with the GI tract. Whereas select animals had detectable virus in the stomach (1 of 7 hFcRn^Tg32^ mice and 2 of 14 hFcRn^Tg32^-IFNLR^−/−^ mice) and large intestine (3 of 14 hFcRn^Tg32^-IFNLR^−/−^ mice), hFcRn^Tg32^-IFNLR^−/−^ animals had higher levels of virus in all regions of the small intestine, including the duodenum (6 of 14 mice), jejunum (6 of 14 mice), and ileum (4 of 14 mice) (Fig. 4A to G). Consistent with the more persistent infection in the GI tract of hFcRn^Tg32^-IFNLR^−/−^ mice, these mice also contained higher levels of virus in the stool (7 of 14 animals with detectable virus) than all other genotypes (Fig. 4F). Male animals contained higher viral titers than did female mice, although these differences were not significant (Fig. S1C). However, even at 7 dpi, we were unable to detect any virus in the blood, liver, pancreas, or brain of any genotype, including hFcRn^Tg32^-IFNLR^−/−^ (Fig. S3A to D). These data show that type III IFNs do not control dissemination but limit persistent infection of the intestine.

10.1128/mbio.00443-22.3 FIG S3 Seven-day-old pups were orally inoculated with 10^6^ PFU of E5. At 7 dpi, animals were sacrificed to measure viral replication in tissues. Viral titers are shown as log_10_ TCID_50_ per milliliter in the blood (A), liver (B), pancreas (C), and brain (D). Data are shown as means ± standard deviations and individual animals (points). Data are shown with significance determined by a Kruskal-Wallis test with Dunn’s test for multiple comparisons (*, *P* < 0.05; **, *P* < 0.005). Each symbol represents an individual animal. Download FIG S3, TIF file, 0.2 MB. Copyright © 2022 Wells et al. 2022 Wells et al. https://creativecommons.org/licenses/by/4.0/ This content is distributed under the terms of the Creative Commons Attribution 4.0 International license.

Visualization of intestinal replication of enteroviruses *in vivo* has been hindered by the lack of sensitive assays to monitor infection with a low signal-to-noise ratio. To overcome this limitation, we utilized HCR, a component of which includes signal amplification, given the self-assembly of secondary detection hairpins into amplification polymers. We inoculated 7-day-old hFcRn^Tg32^-IFNAR^−/−^ and hFcRn^Tg32^-IFNLR^−/−^ animals with 10^6^ PFU of E5 by the oral route, sacrificed them at 3 dpi, and then performed Swiss rolling of full intestinal tissue, which was sectioned and processed for HCR. In contrast to viral titer data, which showed high levels of virus in the intestines of hFcRn^Tg32^-IFNAR^−/−^ animals, we did not detect any vRNA in any intestinal section of these animals (Fig. 4G). However, we observed clear areas of vRNA-containing cells in various regions of the small intestines of hFcRn^Tg32^-IFNLR^−/−^ animals (Fig. 4G). While vRNA was detected in both the duodenum and jejunum, there were more vRNA-containing cells in the ileum of the hFcRn^Tg32^-IFNLR^−/−^ animals (Fig. 4G and H), suggesting that there may be regional differences in echovirus persistence in the epithelium. Although we observed areas of viral replication within the epithelium, we did not see any damage to the epithelium. A pathologist reviewed hematoxylin and eosin (H&E)-stained sections from uninfected, hFcRn^Tg32^-IFNAR^−/−^, and hFcRn^Tg32^-IFNLR^−/−^ animals in a blind manner and observed no significant changes or damage to the intestine following E5 infection at 3 dpi (Fig. S4A). Additionally, we observed no change to the cellular composition of the epithelium as suggested by periodic acid-Schiff (PAS) staining for goblet cells (Fig. S4B).

10.1128/mbio.00443-22.4 FIG S4 Seven-day-old pups were orally inoculated with 10^6^ PFU of E5. At 3 dpi, animals were sacrificed, and intestines were collected for histology. (A) Hematoxylin and eosin staining of representative intestinal sections from uninfected, hFcRn^Tg32^-IFNAR^−/−^, or hFcRn^Tg32^-IFNLR^−/−^ animals. (B) Periodic acid-Schiff staining of representative intestinal sections from uninfected or hFcRn^Tg32^-IFNLR^−/−^ animals to identify goblet cells. Bars, 100 μm. Download FIG S4, TIF file, 1.5 MB. Copyright © 2022 Wells et al. 2022 Wells et al. https://creativecommons.org/licenses/by/4.0/ This content is distributed under the terms of the Creative Commons Attribution 4.0 International license.

### Enterocytes are the main cellular targets of echoviruses *in vivo*.

**FIG 5:**
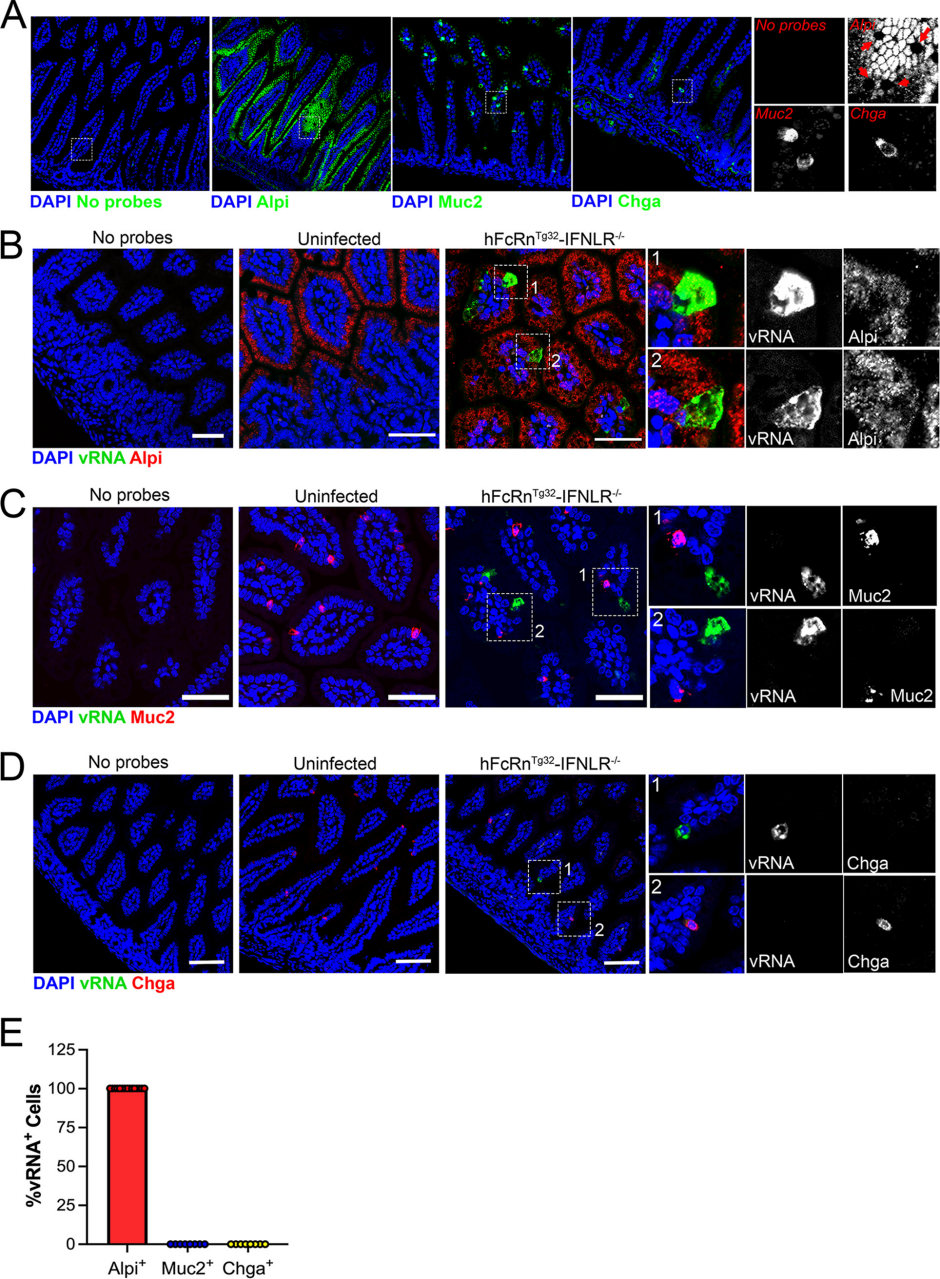
*In vivo* replication of echoviruses is specific for enterocytes. (A) Hybridization chain reaction (HCR) RNA-FISH of uninfected small intestine sections using specific probes against Alpi, Muc2, or Chga (in green), as indicated at the bottom. DAPI-stained nuclei are shown in blue. A no-probe-containing control is shown on the left. In all panels, the white box is shown zoomed in (magnification, approximately ×6) at the right using the probes indicated in red. Red arrows in the Alpi section denote goblet cells based on morphology that were not positive for Alpi, as expected. (B to D) Seven-day-old hFcRn^Tg32^-IFNLR^−/−^ neonatal mice were orally inoculated with 10^6^ PFU of E5; at 3 dpi, animals were sacrificed; and the entire small intestine was removed and Swiss rolled for subsequent histological sectioning. Shown are representative images of ileum tissue using probes for E5 (green in all panels) and either Alpi (B), Muc2 (C), or Chga (D) (red in all panels). DAPI-stained nuclei are shown in blue. In all panels, white boxes denote zoomed-in areas shown on the right, which include black-and-white images, as indicated. Bars, 50 μm. (E) Quantification of confocal images was performed using FIJI and expressed as the total percentage of vRNA-positive cells that colocalized with Alpi (in red), Muc2 (in blue), or Chga (in green). Note that there was no colocalization between vRNA and either Muc2 or Chga.

We showed previously that echoviruses preferentially infect enterocytes and enteroendocrine cells in human stem cell-derived enteroids (9). However, whether there is a cell type specificity of infection for echoviruses, or other enteroviruses, *in vivo* is unknown. To define the cellular tropism of echoviruses *in vivo*, we designed HCR probes targeting an enterocyte marker (alkaline phosphate, intestinal [Alpi]), a goblet cell marker (mucin-2 [Muc2]), and an enteroendocrine cell marker (chromogranin A [Chga]). We confirmed the specificity of these probes in murine-derived intestinal tissue and found that they accurately labeled distinct cell populations in the epithelium (Fig. 5A). Using probes directed against E5 and Alpi, Muc2, or Chga, we performed HCR in Swiss-rolled intestinal tissue sections isolated from hFcRn^Tg32^-IFNLR^−/−^ animals orally infected with 10^6^ PFU of E5 at 3 dpi. We found that echovirus vRNA localized exclusively to Alpi-positive cells (Fig. 5B) and was not observed in any Muc2-positive goblet cells (Fig. 5C) or Chga-positive enteroendocrine cells (Fig. 5D), as assessed by image analysis and quantification of confocal microscopy-generated tile scans of ~4 mm^2^ of intestinal tissue (Fig. 5E; Fig. S5). These data show that enterocytes are the main targets of echoviruses following oral inoculation of hFcRn^Tg32^-IFNLR^−/−^ mice.

10.1128/mbio.00443-22.5 FIG S5 Representative tile scan of an ileum from an hFcRn^Tg32^-IFNLR^−/−^ pup, with vRNA shown in green and DAPI in blue. The tile scan was done at a ×20 magnification, with an area of 6 by 6 tiles combined for a total of 36 individual images that were stitched together. The total area of view is 4 mm^2^ per image. Download FIG S5, TIF file, 1.0 MB. Copyright © 2022 Wells et al. 2022 Wells et al. https://creativecommons.org/licenses/by/4.0/ This content is distributed under the terms of the Creative Commons Attribution 4.0 International license.

## DISCUSSION

The events associated with enterovirus infections of the GI tract *in vivo* are largely unknown. Here, we defined the role of hFcRn and type-specific IFN signaling in mediating echovirus infections of the intestinal epithelium and dissemination to secondary tissue sites. We show that hFcRn is necessary and sufficient for echovirus infection of the intestinal epithelium in enteroids derived from humanized FcRn mice. However, *in vivo*, the expression of hFcRn alone is not sufficient for echovirus infection by the enteral route. Using humanized FcRn mouse models deficient in either type I or III IFN signaling, we defined the differential roles of these IFNs in echovirus replication in and dissemination from the GI tract. These studies showed that type I IFNs limit the dissemination of echoviruses from the GI tract, and ablation of this signaling robustly increases viral replication at secondary sites such as the liver. In contrast, type III IFNs suppress replication in the intestinal epithelium, and deletion of the receptor for these IFNs prolongs intestinal echovirus replication and increases viral persistence. We further show that echoviruses preferentially infect enterocytes within the small intestine *in vivo*, which is enhanced in the absence of type III IFN signaling. Collectively, our work presented here provides key insights into the roles of FcRn and IFN signaling in echovirus pathogenesis in the GI tract.

Little is known regarding the mechanisms used by echoviruses to enter the intestinal epithelium. Our data support a model whereby hFcRn is necessary and sufficient for intestinal replication *in vitro*. While some echoviruses utilize decay-accelerating factor (DAF/CD55) as an attachment factor *in vitro* (25), E5 does not bind DAF (21). Moreover, DAF-binding echoviruses do not bind the murine homolog of DAF (25). While a previous study predicted that echovirus binding to DAF might trigger viral internalization and particle delivery to endosomes, at which time FcRn-mediated uncoating would occur (22), the data presented here do not support such a model and suggest that DAF plays no role in echovirus infections of the intestinal epithelium *in vitro* or *in vivo*. Instead, our data suggest that FcRn is necessary and sufficient for echovirus infection of the intestinal epithelium and occurs independently of DAF binding. This is consistent with *in vivo* data from humanized mouse models of DAF that showed that the expression of DAF does not impact intestinal replication of DAF-binding variants of CVB (29).

FcRn is unique in its ability to mediate the transcytosis of IgG and albumin across the intestinal epithelium. Interestingly, this transport functions in a bidirectional manner in cultured intestinal cell lines, suggesting that FcRn can sample contents from the apical or basolateral domain and mediate the transcytosis of cargo to the opposing domain (30). This function of FcRn could have important implications during echovirus infections: FcRn could (i) mediate the internalization of viral particles into intracellular compartments that facilitate uncoating and subsequent replication and/or (ii) mediate the direct transcytosis of viral particles across the intestinal epithelium from the lumen into underlying tissue. Given that FcRn mediates bidirectional transport across the epithelium, this raises the possibility that echoviruses could be transported from either the apical or basolateral domain to cross the intestinal barrier. We were unable to visualize active replication in the intestinal epithelium of hFcRn^Tg32^-IFNAR^−/−^ animals despite robust viral dissemination to secondary sites of infection. In contrast, we detected vRNA in ~6% of enterocytes in ileum tissues of hFcRn^Tg32^-IFNLR^−/−^ animals, in which there was no dissemination observed. These data suggest that in addition to facilitating viral entry and replication in enterocytes, it is possible that in some cases, FcRn might facilitate the transcytosis of echovirus particles across the epithelium and that ablation of type I IFN signaling promotes the dissemination of these particles to secondary sites of infection.

Type III IFNs are important in the antiviral defenses of many barrier tissues, including the GI tract (31, 32). For example, IFN-λs control rotavirus infection in the intestinal epithelium in adult and neonatal mice (19). This study showed that whereas mice lacking IFNLR were more susceptible to rotavirus replication and virus-induced cytotoxicity, IFNAR^−/−^ mice were comparable to immunocompetent WT mice. These data are distinct from those of our work presented here, which shows that type I IFNs are key host mediators that prevent echovirus dissemination following oral infection. Type III IFNs have also been implicated in restricting murine norovirus replication in the GI tract *in vivo* (18). Similar to our findings with echoviruses, IFN-λs restrict persistent norovirus infection, whereas type I IFNs restrict dissemination (18). Our data suggest that at acute times postinfection, echovirus infection is persistent within the GI epithelium. However, it is possible, and perhaps likely, that following longer periods of time, echovirus can be cleared from the epithelium.

Previous studies with PV and EV71 suggest that type I IFNs control the viral replication of these enteroviruses by the enteral route *in vivo* (12, 13), whereas CVB infection results in only a modest increase in titers in animals deficient in IFNAR (29). While the mechanistic basis for these differences is unknown, it is possible that the cell type-specific nature of enterovirus replication in the intestine may influence their dependence on IFN signaling. For example, in human enteroids, EV71 preferentially infects goblet cells, whereas echoviruses are enriched in enterocytes (9, 10). In cell lines, previous work suggested that PV transcytoses across M cells, suggesting that it does not replicate in the epithelium (33). Future studies on the cell type-specific nature of IFN signaling in distinct lineages of intestinal cells and the impact of these differences on enterovirus replication will be essential to determine if the distinct cellular tropism of enteroviruses in the GI tract influences IFN-mediated signaling.

Interestingly, our *in vitro* data using murine-derived enteroids suggest that while human FcRn expression is sufficient for infection of echoviruses, disruption of type I or III IFN signaling does not contribute to infection in the epithelium. However, we found that this is not the case *in vivo*, in which immunocompetent mice expressing human FcRn were almost completely resistant to infection by the enteral route. In contrast, hFcRn-expressing animals lacking type I and III IFN signaling exhibited viral replication in distinct tissues. Given the significant differences between *in vitro* and *in vivo* models of the GI tract, these differences may not be surprising. For example, enteroids do not recapitulate the diverse immune cell repertoire present in the GI tract, which can impart regional differences in immune defenses (reviewed in reference 34). The presence of immune cells undoubtedly impacts infection by the enteral route and likely imparts protection. Perhaps most notably, enteroids do not recapitulate the microbial landscape of the GI tract, which is known to impact various aspects of enteric virus replication. Previous studies using PV and CVB have shown that the administration of antibiotics prior to oral inoculation reduces viral replication and lethality, suggesting that the microbiota facilitates infection by at least some enteroviruses (15, 35). In fact, specific bacteria within the microbiota can stabilize PV particles by binding to the capsid proteins, which promotes infection within the intestine (36). Other studies have shown that the microbiota stimulates pockets of type III IFN induction within the epithelium of the GI tract, resulting in the upregulation of ISGs that can protect against enteric virus infection (37). Collectively, this work further supports that enterovirus infection of the GI tract involves a complex interplay among the virus, microbiota, and GI-derived cells.

Our findings presented here define fundamental aspects of echovirus biology that enhance our understanding of how infection, tissue targeting, and disease occur *in vivo*. We show that FcRn is necessary but not sufficient for echovirus infections of the GI tract *in vivo* and that type I and III IFNs differentially control echovirus persistence and dissemination. Collectively, these studies provide new insights into echovirus biology and the development of *in vivo* models that recapitulate distinct aspects of echovirus disease, which could potentially accelerate the development of therapies.

## MATERIALS AND METHODS

### Cell lines and viruses.

HeLa cells (clone 7B) were provided by Jeffrey Bergelson, Children’s Hospital of Philadelphia, Philadelphia, PA, and cultured in minimal essential medium (MEM) supplemented with 5% fetal bovine serum (FBS), nonessential amino acids (NEAA), and penicillin/streptomycin (pen/strep). Experiments were performed with echovirus 5 (E5) (Noyce strain), which was obtained from the ATCC. Virus was propagated in HeLa cells and purified by ultracentrifugation over a 30% sucrose cushion, as described previously (38). Enteroid experiments were performed with light-sensitive neutral red (NR)-incorporated viral particles. E5 was propagated in the presence of NR (10 μg/mL) under semidark conditions and subsequently purified under semidark conditions by ultracentrifugation over a sucrose cushion (39). All viral stocks used in the study were sequenced for viral purity following propagation (41). To do this, RNA extraction was performed on 10 μL of the purified virus stock (catalog no. 529904; Qiagen). RNA was reverse transcribed using a SuperScript III reverse transcription kit (catalog no. 18080093; Invitrogen), according to the manufacturer’s instructions, using a panenterovirus primer (vir21) (see Table S1 in the supplemental material), followed by RNase H treatment for 20 min at 37°C. PCR was performed with 5 μL of the cDNA reaction mixture using Bio-Rad iTaq DNA polymerase (catalog no. 1708870). Briefly, each 50-μL reaction mixture consisted of a final concentration of the following components: 1× iTaq buffer; 1.5 mM MgCl_2_; 200 μM deoxynucleoside triphosphate (dNTP); 1.25 U iTaq DNA polymerase; 500 nM forward and reverse primers for a number of enteroviruses, including E5, E11, and CVB, or panenterovirus primers (Table S1); 5 μL of cDNA; and water to a final volume of 50 μL. The PCR program was as follows: 95°C for 3 min; 40 cycles of 95°C for 30 s, a 42°C ramp at 0.4°C/s for 45 s, and 72°C 45 s; 72°C for 5 min; and a 4°C hold. Samples were run on a 1% agarose gel, and the resulting bands were gel extracted using the Thermo GeneJET gel extraction kit (catalog no. K0692). PCR products were confirmed by Sanger sequencing (Genewiz) using the indicated VP1-specific primers mentioned above.

10.1128/mbio.00443-22.6 TABLE S1 VP1 PCR sequencing primers. Download Table S1, DOCX file, 0.01 MB. Copyright © 2022 Wells et al. 2022 Wells et al. https://creativecommons.org/licenses/by/4.0/ This content is distributed under the terms of the Creative Commons Attribution 4.0 International license.

### Animals.

All animal experiments were approved by the Duke University Animal Care and Use Committees. C57BL/6J (WT) (catalog no. 000664), B6.Cg-*Fcgr^t^*^tm1Dcr^Tg(FCGRT)32Dcr/DcrJ (hFcRn^Tg32^) (catalog no. 014565), and B6.(Cg)-Ifnar1^tm1.2Ees^/J (IFNAR^−/−^) (catalog no. 028288) mice were purchased from The Jackson Laboratory. hFcRn^Tg32^-IFNAR^−/−^ mice were generated as described previously (23). B6.Ifnlr^−/−^/J (IFNLR^−/−^) mice were provided by Megan Baldridge (Washington University School of Medicine). hFcRn^Tg32^-IFNLR^−/−^ mice were generated by crossing B6.Cg-*Fcgr^t^*^tm1Dcr^Tg(FCGRT)32Dcr/DcrJ (hFcRn^Tg32^) (catalog no. 014565) mice with B6.Ifnlr^−/−^/J mice. Breeders were established that were deficient in mouse FcRn and IFNLR and were homozygous for the hFcRn transgene. Genotypes of all animals used in this study were confirmed by Transnetyx, and genotyping assays are available upon request.

### Enteroid isolation and passaging.

Murine intestinal crypts were isolated using a protocol adapted from the protocol of Stem Cell Technologies. Briefly, intestines were isolated from five 10-day-old pups, and connective tissue was removed. Intestines were cut longitudinally and washed extensively in phosphate-buffered saline (PBS). Intestines were cut into 5-mm segments and washed again using a 10-mL serological pipette until PBS was clear. Washed intestinal pieces were incubated in a gentle cell dissociation reagent (catalog no. 07174; Stem Cell Technologies) for 15 min at room temperature. Crypts were released using 0.1% bovine serum albumin (BSA) and vigorous pipetting with a 10-mL serological pipette. Crypts were filtered using a 70-μm cell strainer, and the resulting flowthrough was centrifuged at 290 × *g* for 5 min. Pellets were resuspended in Matrigel (catalog no. 356231; Corning), and 40 μL of crypt-containing Matrigel “domes” was plated into each well of a 24-well plate (catalog no. 3526; Corning), placed in a 37°C incubator to prepolymerize for ~3 min, turned upside down to ensure an equal distribution of the isolated cells in domes for another 10 min, and then carefully overlaid with 500 μL IntestiCult organoid growth medium (catalog no. 06005; Stem Cell Technologies) supplemented with 1% penicillin/streptomycin, 50 μg/mL gentamicin, and 0.2% amphotericin B, containing Y-27632 (Rock inhibitor; Sigma). Medium was changed every 48 h, and Y-27632 was removed after the first medium change.

Confluent enteroids were passaged by manual disruption of Matrigel domes with a P1000 pipette tip in PBS and centrifuged at 400 × *g* for 5 min. The enteroid-containing pellets were resuspended in TrypLE (catalog no. 12605010; Invitrogen) and incubated in a water bath at 37°C for 8 min. Enzyme activity was quenched with Dulbecco’s modified Eagle’s medium (DMEM) containing 10% FBS, and the mixture was centrifuged at 400 × *g* for 5 min. Pellets were resuspended in Matrigel and 40 μL of Matrigel domes in each well of a 24-well plate. Domes were allowed to solidify at 37°C for 10 min and then covered with IntestiCult organoid growth medium, as described above. For infections, passaged enteroids were plated into 24-well plates precoated with 15 μL of Matrigel using a P1000 tip and allowed to solidify for 30 min. Enteroids were grown in IntestiCult organoid growth medium and allowed to differentiate for 5 days prior to infections.

### Enteroid infections.

Enteroids plated on Matrigel coating as described above were allowed to differentiate for 5 days, with IntestiCult organoid growth medium being replaced every 48 h. For infections, wells were infected with 10^6^ PFU of NR-incorporated virus, generated as described above. Virus was preadsorbed for 1 h at 16°C, enteroids were washed three times with PBS, and medium was replaced. Infections were initiated by shifting enteroids to 37°C. At 6 hpi, enteroids were exposed to light on a light box for 20 min to render intact viral particles noninfectious, and infections were performed for the times indicated. Plaque assays were performed in HeLa cells overlaid with a 1:1 mixture of 1% agarose and 2× MEM (4% FBS, 2% pen/strep, 2% NEAA). Plaques were enumerated at 40 hpi by crystal violet staining.

### RNA extraction and RNA sequencing.

Total RNA was prepared using the Sigma GenElute total mammalian RNA miniprep kit with an optional DNase step, according to the protocol of the manufacturer. RNA quality was assessed by a Nanodrop instrument and an Agilent RNA Screen Tape system, and 1 μg was used for library preparation using RNA with a poly(A) selection kit (Illumina), according to the manufacturer’s instructions. Sequencing was performed on an Illumina HiSeq platform. RNASeq FASTQ data were processed and mapped to the mouse reference genome (GRCm38) using CLC Genomics Workbench 20 (Qiagen). Differential gene expression was determined using the DESeq2 package in R (40). Heatmaps and volcano plots were made in GraphPad Prism 9. Raw sequencing data were deposited under BioProject PRJNA838604.

### Suckling pup infections.

Seven-day-old mice were inoculated by the oral route with 10^6^ PFU of E5. Oral gavage inoculation was performed using a 1-mL disposable syringe and a 24-gauge round-tipped needle in 50 μL of 1× PBS. Mice were euthanized at either 3 or 7 days postinoculation, and organs were harvested into 0.5 mL of DMEM and stored at −80°C. Tissue samples for viral titration were thawed and homogenized with a TissueLyser LT instrument (Qiagen) for 5 min, followed by brief centrifugation for 5 min at 8,000 × *g*. Viral titers in organ homogenates were determined by a 50% tissue culture infective dose (TCID_50_) assay in HeLa cells and enumerated following crystal violet staining.

### HCR and imaging.

HCR was performed according to the Molecular Instruments HCR v3.0 protocol for formalin-fixed paraffin-embedded (FFPE) human tissue sections (27, 28). Briefly, tissue sections were deparaffinized with xylene and rehydrated with decreasing concentrations of ethanol (100%, 95%, and 80%). Antigen unmasking was performed with slides submerged in 10 mM citrate buffer (pH 6.0) and heated in a steamer for 20 min at ~90°C. Slides were cooled to room temperature. Sections were treated with 10 μg/mL proteinase K for 10 min at 37°C and washed with RNase-free water. Samples were incubated for 10 min at 37°C in hybridization buffer. Sections were incubated overnight in a humidified chamber at 37°C with 3 pmol of initiator probes in hybridization buffer. We designed probes for albumin (23), E5 (Table S2), Muc2 (Table S3), and Chga (Table S4) in-house. Custom probes for Alpi were designed by Molecular Instruments (lot no. PRI910). The next day, slides were washed in probe wash buffer and 5× saline-sodium citrate buffer with 0.1% tween-20 (SSCT) four times for 15 min each, according to the manufacturer’s instructions. Samples were incubated in a humidified chamber at 37°C for 30 min in amplification buffer. Fluorescent hairpins were heated to 95°C for 90 s and snap-cooled at room temperature for 30 min. Hairpins and amplification buffer were added to the sample, and the mixture was incubated overnight at room temperature. Hairpins were washed off with 5× SSCT for 5 min, 15 min, 15 min, and 5 min, followed by a wash with PBS containing 4′,6-diamidino-2-phenylindole (DAPI). Slides were mounted in Vectashield with DAPI. Slides were imaged on a Zeiss 880 with Airyscan inverted confocal microscope. Tile scans were performed at a ×20 magnification using a 6-by-6 square area, resulting in 36 total images. Each intestinal segment was tile scanned using three different areas for quantification. Image analysis was performed using FIJI.

10.1128/mbio.00443-22.7 TABLE S2 Echovirus 5 HCR probes. Download Table S2, DOCX file, 0.01 MB. Copyright © 2022 Wells et al. 2022 Wells et al. https://creativecommons.org/licenses/by/4.0/ This content is distributed under the terms of the Creative Commons Attribution 4.0 International license.

10.1128/mbio.00443-22.8 TABLE S3 Muc2 HCR probes. Download Table S3, DOCX file, 0.01 MB. Copyright © 2022 Wells et al. 2022 Wells et al. https://creativecommons.org/licenses/by/4.0/ This content is distributed under the terms of the Creative Commons Attribution 4.0 International license.

10.1128/mbio.00443-22.9 TABLE S4 Chga HCR probes. Download Table S4, DOCX file, 0.01 MB. Copyright © 2022 Wells et al. 2022 Wells et al. https://creativecommons.org/licenses/by/4.0/ This content is distributed under the terms of the Creative Commons Attribution 4.0 International license.

### Periodic acid-Schiff staining.

Periodic acid-Schiff (PAS) staining was performed according to the manufacturer’s instructions (catalog no. ab150680; Abcam). Slides were mounted with Cytoseal 60 (catalog no. 83104; Thermo Scientific). Images were captured on an IX83 inverted microscope (Olympus) using a UC90 color charge-coupled-device (CCD) camera (Olympus).

### Statistics.

All statistical analysis was performed using GraphPad Prism version 8. Data are presented as means ± standard deviations (SD). One-way analysis of variance (ANOVA) was used to determine statistical significance, as described in the figure legends. Parametric tests were applied when data were distributed normally based on D’Agostino-Pearson analyses; otherwise, nonparametric tests were applied. *P* values of <0.05 were considered statistically significant, with specific *P* values noted in the figure legends.
