# Supplementary figures and images for: Enterovirus Replication and Dissemination Are Differentially Controlled by Type I and III Interferons in the Gastrointestinal Tract
Source: mBio. 2022 May 23;13(3):e00443-22. doi: 10.1128/mbio.00443-22 (PMC9239134; doi:10.1128/mbio.00443-22)

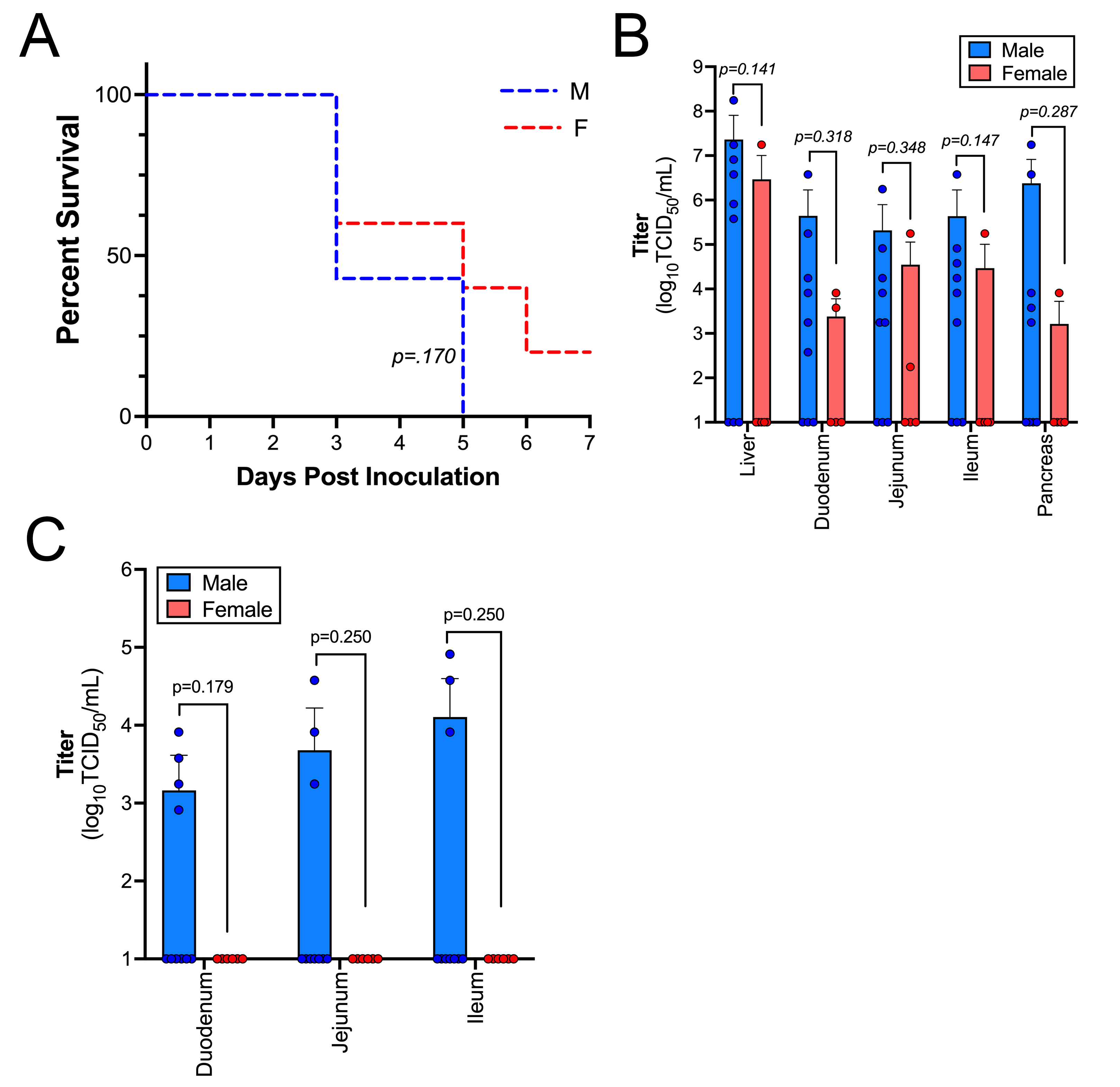

Supplement: FIG S1 [file mbio.00443-22-s0001.tif]

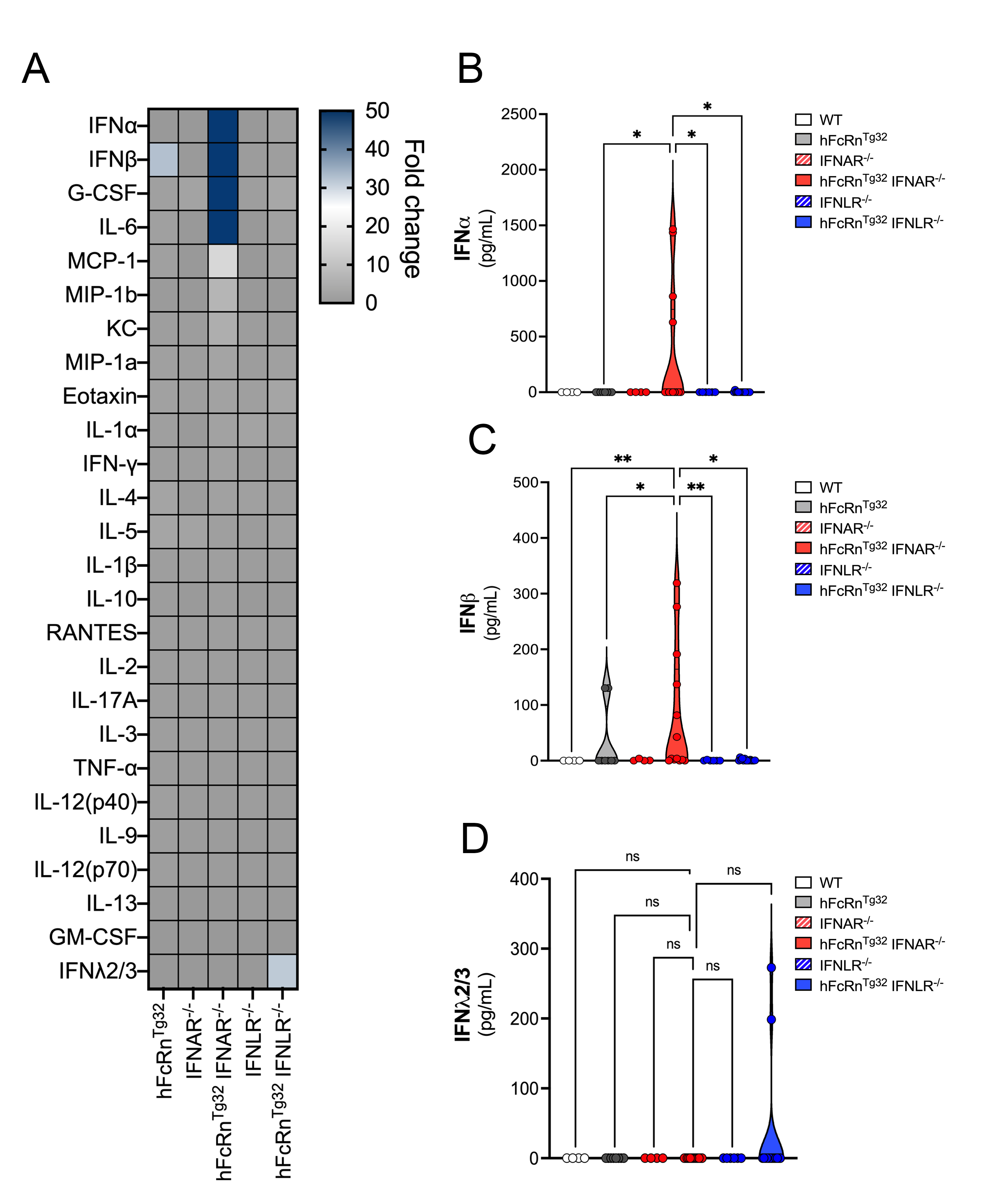

Supplement: FIG S2 [file mbio.00443-22-s0002.tif]

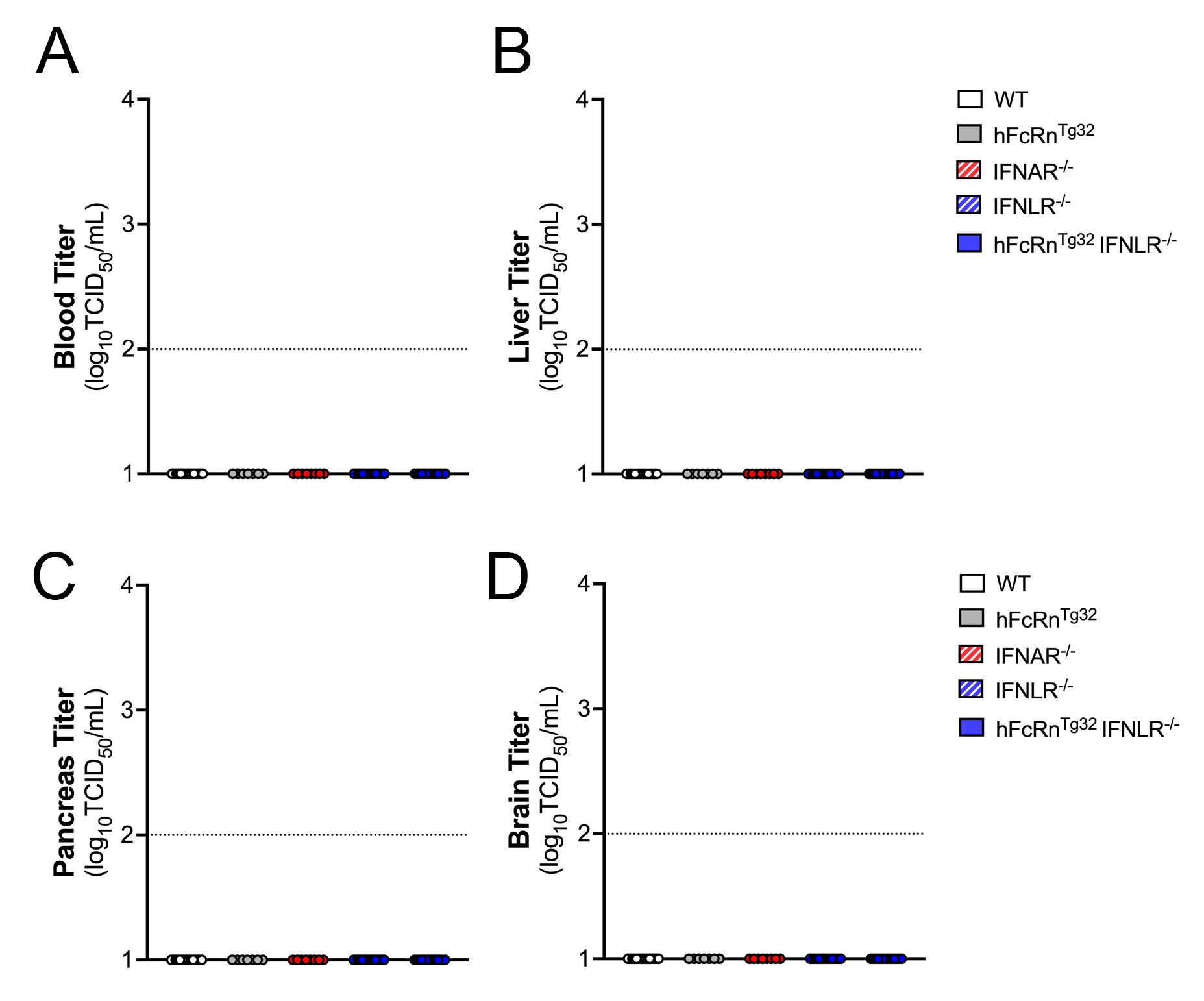

Supplement: FIG S3 [file mbio.00443-22-s0003.tif]

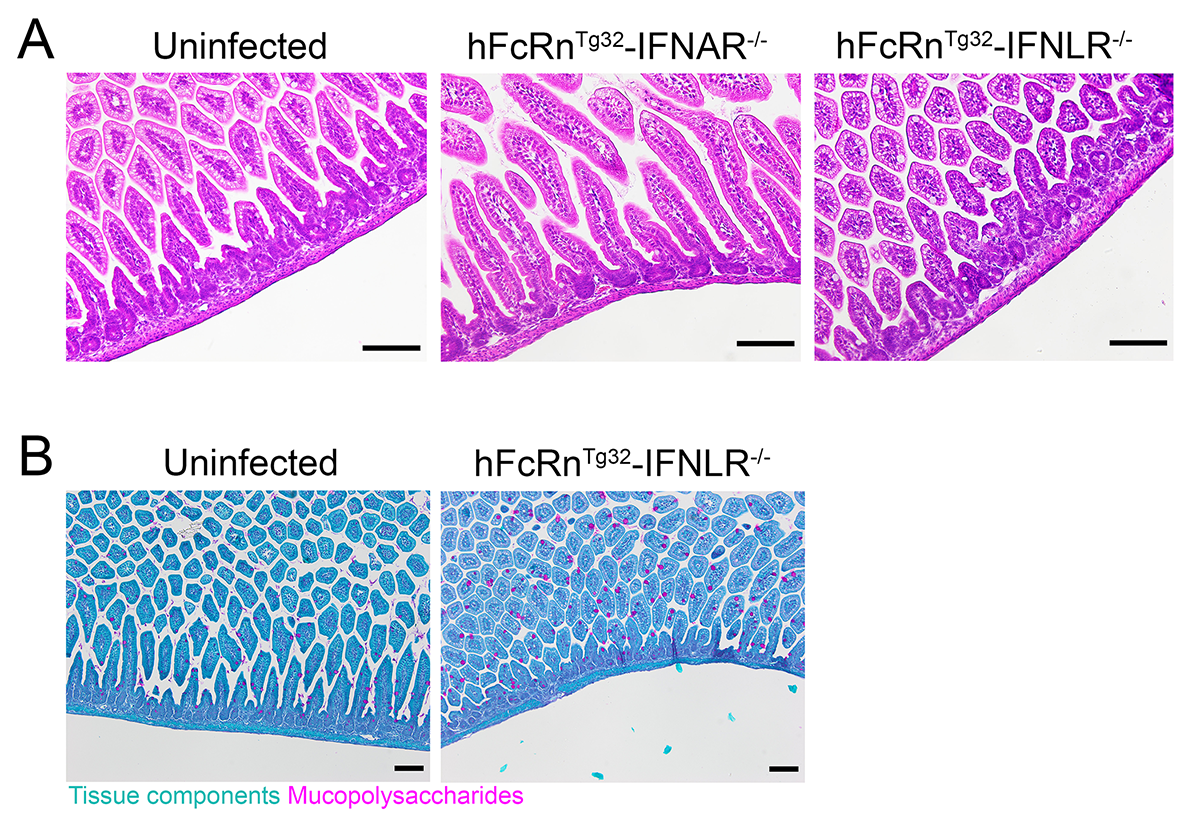

Supplement: FIG S4 [file mbio.00443-22-s0004.tif]

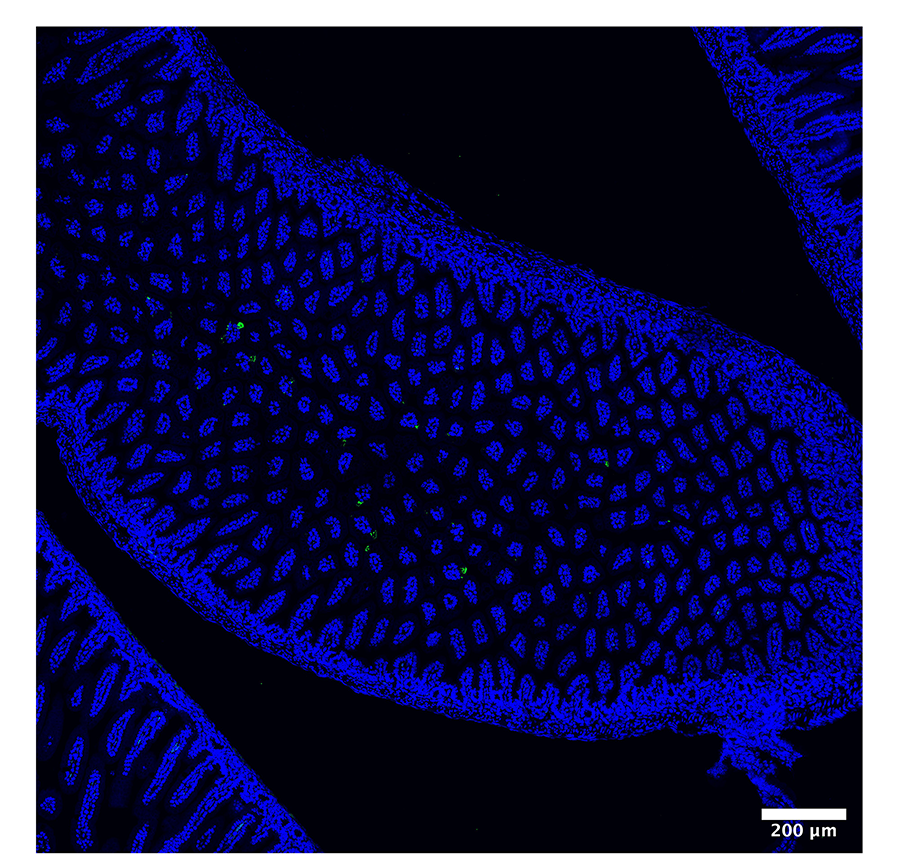

Supplement: FIG S5 [file mbio.00443-22-s0005.tif]
