# Supplementary material for: Enterovirus Replication and Dissemination Are Differentially Controlled by Type I and III Interferons in the Gastrointestinal Tract
Source: mBio. 2022 May 23;13(3):e00443-22. doi: 10.1128/mbio.00443-22 (PMC9239134; doi:10.1128/mbio.00443-22)
Supplement: TABLE S1 [file mbio.00443-22-s0006.docx]

| **Target** | **Forward Primer 5’-3’** | **Reverse Primer 5’-3’** |
| --- | --- | --- |
| vir21 | ATAAGAATGCGGCCGCTTTTTTTTTT  TTTTTTTTTTTTTTT | N/A |
| Pan_VP1 | MIGCIGYIGARACNGG | CICCIGGIGGIAYRWACAT |
| E5_VP1 | TATCGCCAATTACAACGCGAA | TTGGTTTGAAGTAAACCCTTA |
| E11_VP1 | CCGTCAAATTCGCAAGCAGT | ACCATGCGTCGTGCACTAAT |
| CVB_VP1 | AGTGGGAACTGGACCGACTA | CCCATTCGGCATAGCGTTTG |
| EV71_VP1 | CTTAACTCGCACAGCACAGC | GGAATCTGGCTTAGGGGCTC |

**Supplemental Table 1.** Primers used for amplification and sequencing of enterovirus stocks.
