# Supplementary material for: Enterovirus Replication and Dissemination Are Differentially Controlled by Type I and III Interferons in the Gastrointestinal Tract
Source: mBio. 2022 May 23;13(3):e00443-22. doi: 10.1128/mbio.00443-22 (PMC9239134; doi:10.1128/mbio.00443-22)
Supplement: TABLE S2 [file mbio.00443-22-s0007.docx]

**Supplemental Table 2.** HCR probes for E5.

| Probe Pair |  |  |
| --- | --- | --- |
| B4P1 | CCTCAACCTACCTCCAACAAGGGCTCAGTAAACTTTCCCGGGTCT | CATGGATTTGATCATAAGGTCCTTCATTCTCACCATATTCGCTTC |
| B4P2 | CCTCAACCTACCTCCAACAAAGCCCTGATGGAACTTAGATGCGTT | CTTCAGGCACACACACCACCAGGAGATTCTCACCATATTCGCTTC |
| B4P3 | CCTCAACCTACCTCCAACAAGCATTACTATGGTGGCACAATTGTT | TGTCCATCGGTACGCTATTAATGTAATTCTCACCATATTCGCTTC |
| B4P4 | CCTCAACCTACCTCCAACAATGCTGCACCATCCTATAGTGGGTTT | ATGTAGCCTGCTGCTGAGTATTCATATTCTCACCATATTCGCTTC |
| B4P5 | CCTCAACCTACCTCCAACAAGTTATGCTCAGACTTGGGCACGTCA | TTGGCCAAAGGCTCCATATGTGTGCATTCTCACCATATTCGCTTC |
| B4P6 | CCTCAACCTACCTCCAACAAGTGATCAGGTCATCGTGATTTCTTA | CCAATGAGGGCTAGTGTGGCAGTCAATTCTCACCATATTCGCTTC |
| B4P7 | CCTCAACCTACCTCCAACAAGATCTTCCAATTAGATTTGTTGCTA | ACTGAGCTGTTAAGCTTTTCAGCTAATTCTCACCATATTCGCTTC |
| B4P8 | CCTCAACCTACCTCCAACAACTCCCTGTATAACGGTGGTCCCTGA | TGTCTCTGGTGCAACACTAATTTTGATTCTCACCATATTCGCTTC |
| B4P9 | CCTCAACCTACCTCCAACAATTAGTGGGCGTGCCGCCCAGGTTTA | GTGGGGAAGTTATACATGAGCATTCATTCTCACCATATTCGCTTC |
| B4P10 | CCTCAACCTACCTCCAACAAAGCGCTTCAAGACCCTCAGTACCGT | TGGGTAACCGGCGCTCGTTGTTAGATATTCTCACCATATTCGCTTC |
| B4P11 | CCTCAACCTACCTCCAACAATGCACAAGTAGTCAATGTAGTTAGT | GCTTGTCTCTGTACAGGTGATGGGAATTCTCACCATATTCGCTTC |
| B4P12 | CCTCAACCTACCTCCAACAATTTCACGAATGTTGATCTCCCACCT | TCTCACACTTTCTACGTAGTTGCACATTCTCACCATATTCGCTTC |
