# Supplementary material for: Enterovirus Replication and Dissemination Are Differentially Controlled by Type I and III Interferons in the Gastrointestinal Tract
Source: mBio. 2022 May 23;13(3):e00443-22. doi: 10.1128/mbio.00443-22 (PMC9239134; doi:10.1128/mbio.00443-22)
Supplement: TABLE S3 [file mbio.00443-22-s0008.docx]

**Supplemental Table 3.** HCR probes for MUC2.

| Probe Pair |  |  |
| --- | --- | --- |
| B1P1 | GAGGAGGGCAGCAAACGGAAGAGAGGCCGGCCCGAGAGTAGACCT | AGTGCATCTTCCCGGTTCCACATGATAGAAGAGTCTTCCTTTACG |
| B1P2 | GAGGAGGGCAGCAAACGGAACGCAGGGCGAGCTGCTCTCCAGGTA | TAACCTCCAGATGTGAGCATGTGTCTAGAAGAGTCTTCCTTTACG |
| B1P3 | GAGGAGGGCAGCAAACGGAAAGTCTTCAGGCAGGTCTGCTTGTCT | CTTCTTGTCGTCAGTCAACAGCACGTAGAAGAGTCTTCCTTTACG |
| B1P4 | GAGGAGGGCAGCAAACGGAAGGAACACCAGTGCTCAGCGTAGTTG | AAAGGGCGTCTCTGACCTCTTCAGGTAGAAGAGTCTTCCTTTACG |
| B1P5 | GAGGAGGGCAGCAAACGGAAATACACTCAGTATGGTAATAGCCAG | AGTCCATCGGGACACACACAGCCACTAGAAGAGTCTTCCTTTACG |
| B1P6 | GAGGAGGGCAGCAAACGGAAAGGTACTGACCCACTTCCCGTGTGA | ATGATGCCGGAGCTGGCTTCCACCATAGAAGAGTCTTCCTTTACG |
| B1P7 | GAGGAGGGCAGCAAACGGAAGAACTCCCAGTAGCAGAAGATACCA | TTGCCCCACTGTTCCATTGGGGCCGTAGAAGAGTCTTCCTTTACG |
| B1P8 | GAGGAGGGCAGCAAACGGAATTAATGGGGTGGTTGGTGAAGTAGT | TTGTTGGTGAGGTGGTTGGTGAGGTTAGAAGAGTCTTCCTTTACG |
| B1P9 | GAGGAGGGCAGCAAACGGAAAGATGGTTGGTGAGGTGGTTGAAGG | TAGGAGAGATGGTTGATGTTGTTATTAGAAGAGTCTTCCTTTACG |
| B1P10 | GAGGAGGGCAGCAAACGGAAGTGAACTGGTTGATGGAGTGGTAGG | TTGGTGAGGTGGTTGATGGGGTGGTTAGAAGAGTCTTCCTTTACG |
| B1P11 | GAGGAGGGCAGCAAACGGAAAGGCACGAAGGCGTGGCACTGGGA | CAGGCAAGCTTCATAGTAGTGCTTGTAGAAGAGTCTTCCTTTACG |
| B1P12 | GAGGAGGGCAGCAAACGGAATACAGGGCACATGGGTACAGGAGAT | GCTCAAAGCCAGAGCTGCAGGAGATTAGAAGAGTCTTCCTTTACG |
