# Supplementary material for: Enterovirus Replication and Dissemination Are Differentially Controlled by Type I and III Interferons in the Gastrointestinal Tract
Source: mBio. 2022 May 23;13(3):e00443-22. doi: 10.1128/mbio.00443-22 (PMC9239134; doi:10.1128/mbio.00443-22)
Supplement: TABLE S4 [file mbio.00443-22-s0009.docx]

**Supplemental Table 4.** HCR probes for CHGA.

| Probe Pair |  |  |
| --- | --- | --- |
| B1P1 | GAGGAGGGCAGCAAACGGAAGCAATGCTATGCCGGCTTTTATATA | ATGGTGGCGGTGGCGGCGGCAGCAGTAGAAGAGTCTTCCTTTACG |
| B1P2 | GAGGAGGGCAGCAAACGGAAGAAAGAGTGGACGAGCTGCTGCAGG | GGAGCGCATAGCGAGCCGGACGGTGTAGAAGAGTCTTCCTTTACG |
| B1P3 | GAGGAGGGCAGCAAACGGAAGTCGGAGATGACTTCCAGGACGCAC | AGGCATGGGGCTGGGTTTGGACAGCTAGAAGAGTCTTCCTTTACG |
| B1P4 | GAGGAGGGCAGCAAACGGAAGCTGCTGCTGCTGCTGCTGCTGCT | TCAAAGCTGCTGTGTTGCTGCTCCTTAGAAGAGTCTTCCTTTACG |
| B1P5 | GAGGAGGGCAGCAAACGGAATCTTGGTTAGGCTCTGGAAAGGCCT | GACTCACTGTCTCCCATCATGGGGGTAGAAGAGTCTTCCTTTACG |
| B1P6 | GAGGAGGGCAGCAAACGGAACCTTCTCTCTAGCCACAGCCTCCTC | CTGCAGTGGGGACTTCTTCAGGCCCTAGAAGAGTCTTCCTTTACG |
| B1P7 | GAGGAGGGCAGCAAACGGAAGGTCCCTACCATGGCCTCTTCCCCA | CCCGCCTTGGGGGAAGAGACCTTGGTAGAAGAGTCTTCCTTTACG |
| B1P8 | GAGGAGGGCAGCAAACGGAACGGGTCCGGAAGGAGAGCTTCATGG | GGCCCAGGATCTCTGAAGCCATAGGTGAAGAGTCTTCCTTTACG |
| B1P9 | GAGGAGGGCAGCAAACGGAAGCTCTCTAGCTCCTGGTCCTCTGCT | CTCCAGCTCTGCCTCGATGGCTGACTAGAAGAGTCTTCCTTTACG |
| B1P10 | GAGGAGGGCAGCAAACGGAAAAGTGTCCAGGGCAGGGGCTGAGAA | GTGCTGACATTCAGGGCTGCCCTGCTAGAAGAGTCTTCCTTTACG |
